# Increased Circulatory Asymmetric Dimethylarginine and Multiple Organ Failure: Bile Duct Ligation in Rat as a Model

**DOI:** 10.3390/ijms15033989

**Published:** 2014-03-05

**Authors:** Jiunn-Ming Sheen, Yu-Chieh Chen, You-Lin Tain, Li-Tung Huang

**Affiliations:** 1 Department of Pediatrics, Kaohsiung Chang Gung Memorial Hospital and Chang Gung University College of Medicine, Kaohsiung 833, Taiwan; E-Mails: ray.sheen@gmail.com (J.-M.S.); gesicht27@gmail.com (Y.-C.C.); 2 Graduate Institute of Clinical Medical Sciences, Chang Gung University College of Medicine, Kaohsiung 833, Taiwan; 3 Department of Traditional Chinese Medicine, Chang Gung University, Linkou 244, Taiwan

**Keywords:** asymmetric dimethylarginine, bile duct ligation, cholestasis, oxidative stress, multiple organ failure

## Abstract

Bile duct ligation (BDL)-treated rats exhibit cholestasis, increased systemic oxidative stress, and liver fibrosis, which ultimately lead to liver cirrhosis. Asymmetric dimethylarginine (ADMA) is a competitive inhibitor of nitric oxide synthase that can decrease the synthesis of nitric oxide. BDL rats have higher plasma and hepatic ADMA levels, which may be due to increased hepatic protein arginine methyltransferase-1 and decreased dimethylarginine dimethylaminohydrolase expression. BDL rats also exhibit renal and brain damage characterized by increased tissue ADMA concentrations. The increased plasma ADMA levels and multiple organ damages seen here are also observed following multiple organ failures associated with critical illness. This review discusses the dysregulation of ADMA in major organs in BDL rats and the role of increased ADMA in multiple organ damages.

## 1. Introduction

Asymmetric dimethylarginine (ADMA) is a naturally occurring amino acid that can competitively inhibit nitric oxide synthase (NOS) to decrease the synthesis of nitric oxide (NO) [1–4]. ADMA can be detected even in neonates. Vida *et al.* have demonstrated that venous cord blood ADMA levels are markedly elevated (~1.06 μM) and fall significantly to almost reach the normal adult levels by postnatal day 2 (~0.66 μM) [5]. In children, plasma ADMA levels are higher than those in adults, and gradually diminish from birth until around 25 years of age, with a mean decrease of 15 nM per year [6–8]. A healthy adult produces 300 μmol (~60 mg) of ADMA per day [9]. Bode-Bogers *et al.* found a significant increase in plasma levels of ADMA in subjects older than 70 years [10].

By inhibiting NO bioavailability, ADMA causes endothelial dysfunction, vasoconstriction, blood pressure elevation and atherosclerosis [11–16]. Increasing evidence reveals that elevated ADMA is associated with many diseases such as peripheral arterial disease, coronary artery disease, preeclampsia, hypertension, stroke, heart failure, chronic kidney disease, portal hypertension in cirrhosis, diabetes mellitus, and insulin resistance in essential hypertension patients [11,13,14,16–20].

## 2. Asymmetric Dimethylarginine (ADMA) Metabolism

There is a range of substrate proteins for type 1 protein arginine methyltransferase (PRMT), and the enzymes and substrates are distributed throughout the whole body. These proteins are largely found in the nucleus and are implicated in the regulation of RNA processing and transcriptional control [21]. Protein-incorporated ADMA is formed by the PRMTs; two methyl groups are added onto one of the terminal nitrogen atoms of the guanidine group of arginine in proteins. Free ADMA is released after proteolysis, thus factors resulting in increased proteolysis will increase the amount of generated ADMA. Two other derivatives that are methylated by PRMTs are symmetric dimethylarginine (SDMA) and monomethylarginine. These two derivatives are produced at 20%–50% of the amount of ADMA [22]. Free ADMA can be transported in or out of cells via the cationic amino acid transporter (CAT) family [11,21–25]. The CATs are the main determinant of the ADMA distribution between the cytosol and the extracellular fluid, and include the CAT-1, CAT-2A, CAT-2B, CAT-3, and CAT-4 isoforms [25].

While ADMA is widely present, the liver and kidney are the major sites of ADMA production, and this is regulated in a dose-dependent manner by l-arginine [26]. Lung is also a major source of ADMA production. The concentration of protein-incorporated ADMA in the lung is almost 4 times higher than those in the liver, kidney, or heart [27]. Wang *et al.* reported that l-arginine can regulate ADMA metabolism by inhibiting the activity of enzyme, dimethylarginine dimethylaminohydrolase (DDAH) [28]. The metabolic regulation of l-arginine and ADMA provides a stable ratio between these two variables and this then ensures NO homeostasis [26].

Excess plasma ADMA can be transported to major organs for ADMA degradation, mostly by the kidney and liver. In humans, approximately 20% of ADMA is excreted by the kidneys into the urine and this ratio is less in rat [29], whereas 80% of ADMA is metabolized by DDAH to l-citrulline and dimethylamine [25].

## 3. ADMA Regulation in Normal Liver Function

One landmark study of the liver in the metabolism of ADMA was published in 1977 by Carnegie and colleagues [30]. They found that patients with liver disease had a significantly decreased urinary ratio of SDMA to ADMA due to increased excretion of ADMA. Since they could not measure the plasma ADMA levels at the time, it was not possible to examine the exact role of the liver in ADMA elimination in their study [30].

Nijveldt *et al.* demonstrated that the liver had a major role in the regulation of plasma ADMA [31]. This group designed an organ balance study in a rat model to assess arteriovenous concentration differences, together with blood flow measurement using radiolabeled microspheres. They found that the liver took up high amounts of ADMA (0.89 nmol/100 g body weight/min) and that SDMA was barely affected by the liver. Based on the calculation of net organ fluxes and fractional extraction rates, the hepatic ADMA extraction was estimated at 4135 ± 480 nmol/day [31]. This study showed that daily hepatic ADMA extraction is ~700 times more than the amount of plasma ADMA in plasma [31].

## 4. Increased Circulatory and Hepatic ADMA Concentrations in the Context of Liver Dysfunction

Hepatocytes take up large amounts of l-arginine from the hepatic circulation, and liver dysfunction is associated with high plasma l-arginine levels [32]. Although fractional extraction of ADMA is slightly higher in the kidney than in the liver, the liver clears more ADMA from the circulation than the kidney because it has a higher total plasma flow [33]. Therefore, the preservation of hepatic clearance of ADMA is a major determinant of circulatory ADMA concentration and liver dysfunction may result in the accumulation of circulatory ADMA despite the compensation from other organs, such as kidney. It is conceivable that specific hepatic abnormalities may have different effects on DDAH expression or activity [31]. This is supported by other findings by Nijveldt *et al.* in patients undergoing major hepatectomy, they showed that the levels of ADMA were increased post-operatively and that ADMA levels were markedly elevated when liver function was severely impaired [34].

In parallel, Mookerjee *et al.* measured ADMA levels and several cytokines in patients suffering from acute liver failure [35]. ADMA levels in the plasma were considerably higher in acute liver failure patients compared with controls [35]. Similarly, patients suffering from decompensated alcoholic cirrhosis exhibited significantly higher plasma ADMA and NO*_x_* (nitrate plus nitrite) concentrations compared with patients suffering from compensated alcoholic cirrhosis, or healthy volunteers [36].

## 5. The Role of Increased Circulatory ADMA in Multiple Organ Failure in Critical Illness

Using a rabbit model of critical illness, Davids and colleagues showed plasma ADMA was significantly correlated with ADMA levels in the liver [37]. Nijveldt *et al.* proposed the so-called ADMA-multiple organ failure hypothesis (MOF) [38]; in critically ill patients, they demonstrated that hepatic function parameters independently correlated with ADMA concentration, which provides further evidence for the hypothesized role of the liver. The ADMA-MOF hypothesis offers an explanation for the association between high plasma ADMA concentrations and adverse outcome. The pathological changes in multiple organ failure such as deterioration of organ blood flow and endothelial damage can be largely ascribed to the local effects of ADMA. The central role of the liver in the ADMA-MOF hypothesis is in line with studies ascribing the prominent role of hepatic dysfunction in the clinical course of critical illness. This observation is supported by other clinical syndromes associated with hepatic failure, such as hepatorenal syndrome (HRS) and hepatic encephalopathy (HE). In these conditions, the primary role of liver dysfunction with secondary organ failure is evident [38].

## 6. Pathogenic Mechanisms of ADMA in Cell and Organ Metabolism

Following depletion of tetrahydrobiopterin (BH_4_), ADMA stimulates superoxide anion (O_2_^−^) production by an uncoupled endothelial NOS (eNOS) [39,40]. Oxidative stress can oxidize BH_4_ to dihydrobiopterin, which uncouples eNOS. ADMA uncouples NOS [40,41], and thereby increases the expression of inflammatory genes. On the other hand, inflammatory genes activate the PRMTs and inhibit the DDAHs [42] resulting in increased levels of ADMA [43]. ADMA inhibits eNOS activity by competing with l-arginine for binding sites on this enzyme and leads to vasoconstriction, increased platelet aggregation [44], increased cell adhesion to the endothelium, increased vascular leakage, and increased vascular smooth muscle cell proliferation [45]. The above-mentioned factors could work together and contribute to the impairment of organ perfusion associated with increased ADMA.

## 7. Bile Duct Ligation (BDL)-Induced Liver Damages in Rat

The BDL model in rat has been used widely to study cholestatic liver injury with associated oxidative stress and fibrogenesis. Developing and adult rats with BDL have elevated serum levels of aspartate aminotransferase, alanine aminotransferase, gamma-glutamyltranspeptidase, bilirubin, alkaline phosphatase, and lactic dehydrogenase [46–50]. BDL in rat stimulates the proliferation of biliary epithelial cells and hepatocyte progenitors, resulting in proliferating bile ductules with accompanying portal inflammation and fibrosis. Cholangiocyte proliferation is initiated after BDL at the edge of the portal tract. Obstructive jaundice occurred in 2 weeks and progressed to cirrhosis in 4 to 6 weeks [51]. Liver fibrosis is characterized by higher histologic activity index scores as well alpha-smooth muscle actin and transforming growth factor β-1 levels that ultimately cause liver cirrhosis [52,53]. The results are similar in mice and rat [54,55]. In a temporal progression pattern, pathological changes in the liver of the rat are more severe at 4 weeks after BDL than at 2 weeks [56]. The developing BDL rat also exhibits a similar trend of liver pathology progression [57].

## 8. Plasma ADMA, Symmetric Dimethylarginine (SDMA) and l-Arginine Concentrations in the BDL Rat

The BDL rat has higher plasma ADMA, SDMA, and l-arginine levels than control rats [57,58]. The ADMA/l-arginine ratios were higher in BDL rats than in sham rats [50,59]. As the disease progresses, 2 and 4 weeks after BDL, rats have higher plasma ADMA, lower plasma l-arginine levels, and a higher ADMA/l-arginine ratio (an index of NO bioavailability) [60], than the sham rat. However, there was no significant difference between rats 2 and 4 weeks after BDL in terms of plasma ADMA levels [57].

## 9. Possible Role of Increased Circulatory ADMA in the Multiple Organ Damages Observed in the BDL Rat

**Figure 1.**
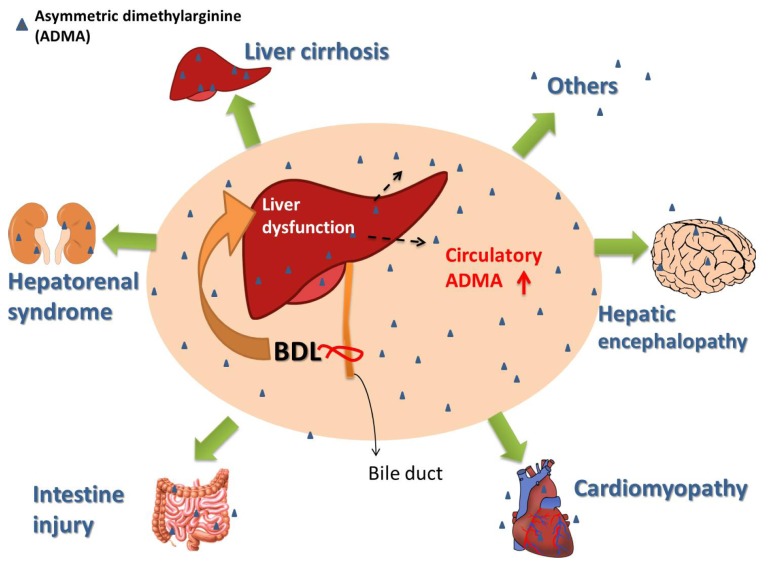
The role of increased circulatory asymmetric dimethylarginine (ADMA) in multiple organ damages in the bile duct ligation rat.

Since the liver is a key organ regulating plasma ADMA concentrations, it is evident that hepatic dysfunction encountered in the BDL rat may disturb ADMA metabolism. The effects of BDL in rat are characterized by increased systemic oxidative stress and damage to major organs, including liver, brain, heart, intestine, and kidney [57,61–63]. In BDL rat, multiple organ damages is related to increased ADMA, an agent known to have pro-oxidant activity. In parallel, cholestatic liver disease is associated with the enhanced generation of reactive oxygen species and increased oxidative stress [50,61]; this increased oxidative stress is a systemic phenomenon encompassing all tissues and organs [50,62–64]. The underlying mechanisms of increased systemic oxidative stress in the BDL rat are complex, involving the intra-organ generation of reactive oxygen species and circulatory toxins, such as bile acid, malondialdehyde, and ADMA [61,65]. Taken together, it is reasonable to assume that increased plasma ADMA may affect multiple organs in the BDL rat. Therefore, BDL in rats can represent a model of increased circulatory ADMA following liver dysfunction with resultant multiple organ damage. Figure 1 depicts the liver dysfunction in the context of BDL and increased circulatory ADMA with the possible role of ADMA in multiple organ damage.

### 9.1. ADMA and Nitric Oxide (NO) Dysregulation in the BDL Rat Liver

Liver cirrhosis is a frequent consequence of the long clinical course of all chronic liver diseases and is characterized by tissue fibrosis and the conversion of normal liver architecture into structurally abnormal nodules. Portal hypertension results from an increased intrahepatic resistance combined with increased portal (and hepatic arterial) blood flow [66]. The increased intrahepatic resistance is the result of architectural distortion (fibrous tissue, regenerative nodules), endothelial dysfunction leading to intrahepatic vasoconstriction, and intrahepatic vascular shunts between afferent and efferent vessels of the liver [67,68].

Dysfunction of sinusoidal endothelial cells in liver cirrhosis is linked with the low production of vasodilators such as NO [69,70]. The activity of eNOS in the cirrhotic liver of humans and rats is significantly decreased [71,72]. In contrast, the concentration of NO*_x_* in the portal venous plasma of patients with cirrhosis and portal hypertension is three-fold higher than that in non-cirrhotic patients, [71] suggesting that NO release is enhanced in the splanchnic vessels of these patients. The difference might be due to a different regulation of eNOS in the liver and in the splanchnic vessels. In terms of inducible NOS (iNOS), its mRNA and protein expressions were intensly induced and were mainly localized in hepatocytes in BDL rat [72]. In human cirrhotic liver, the iNOS was highly expressed in the inflammatory cells infiltrating the portal tracts, blood monocyte-like cells, hepatocytes, sinusoidal cells, and endothelial cells [73].

Animal studies also demonstrated increased plasma and hepatic levels of ADMA in the cirrhosis adult rat [18,58]. In line with previous reports, our data showed that the plasma ADMA level was increased in the BDL developing rat [57,65]. In parallel, in rats with thioacetamide-induced cirrhosis, decreased eNOS enzyme levels seem to be responsible for impaired NOS activity. In rat with bile duct excision-induce biliary cirrhosis, ADMA mediates the decreased NOS activity [18].

Serna *et al.* showed that basal release of NO is increased in small mesenteric arteries of rats with secondary biliary cirrhosis and that the ADMA/DDAH pathway was involved in the increased generation of endothelial NO [74]. In mesenteric vessels, the increased DDAH-1 and DDAH-2 acted to protect NOS enzymes from the increased plasma ADMA levels associated with cirrhosis [74]. Previous research had showed that the expression of hepatic PRMT1 was increased in the BDL developing rat [50], yet the hepatic protein expression of DDAH-1 and DDAH-2, and DDAH activity were unaltered [50,59,65]. The hepatic CAT-1 protein level was increased in the BDL rat [75], while the expression of CAT-2 was decreased [76]. ADMA metabolism is at the whole body level. So, some organs may use CATs to export ADMA to the plasma compartment and other organs may serve as a sink for ADMA influx. It is not surprising to find complex patterns of CATs regulation in different situations.

Yang *et al.* administered vitamin E to decrease lipid peroxidation in the BDL adult rat and reported the suppression of hepatic thiobarbituric acid reactive substances and type 1 protein arginine *N*-methyltransferase (PRMT-1), and increased DDAH-2, eNOS, phospho-eNOS, and ADMA levels in the cirrhotic liver [58]. Tain *et al.* also reported that melatonin decreased liver injury in BDL rats by reducing the level of ADMA (by increasing DDAH activity) and oxidative stress [50].

In parallel, plasma ADMA is also increased in cirrhosis patients. Vizzutti *et al.* investigated the relationship of ADMA in patients with compensated cirrhosis [77]. They found that ADMA may play a pathophysiological role in portal hypertension by contributing to the relative intrahepatic NO deficiency typical of endothelial dysfunction [77]. Lluch *et al.* demonstrated that patients with decompensated alcoholic liver cirrhosis had higher plasma ADMA and NO*_x_* levels than patients with compensated liver cirrhosis and the control group [36]. Increased liver ADMA level could also contribute to impaired endothelium-dependent vasodilation and insulin resistance in a group of hypertensive patients with liver steatosis [78].

The Child-Pugh score is used to evaluate the severity of liver disease, with class C being the most severe, followed by B, and then by A [79]. Plasma ADMA levels were found to be higher in liver cirrhosis patients with Child-Pugh score B compared with patients with Child-Pugh score A [80]. Model for End-Stage Liver Disease (MELD) is a scoring system used to predict survival in cirrhosis patients [81]. The plasma ADMA levels in the cirrhosis group were reported to significantly correlate with MELD scores, but not with age or the ammonia level [82]. Mookerjee *et al.* also examined the levels of plasma ADMA, SDMA, and their combined sum in patients with liver cirrhosis, with or without alcoholic hepatitis, and found that they were all better predictors of outcome compared with the Child-Pugh score, MELD and Maddrey’s discriminant-function [83], an index used to predict prognosis in patients with alcoholic hepatitis [84]. The ADMA/l-arginine ratio was higher in patients with modest/massive ascites compared with the patients with no/little ascites [80]. Siroen *et al.* analyzed the change of dimethylarginine plasma levels in cirrhotic patients receiving a transjugular intrahepatic portosystemic shunt (TIPS) and found that the ratio of ADMA/l-arginine decreased after TIPS placement and suggested an increase in intracellular NO bioavailability [85].

### 9.2. ADMA and NO Dysregulation in the BDL Rat Kidney

The BDL rat exhibits renal damage presenting as higher creatinine levels and elevated tubulointerstitial injury scores compared to those in the control [50,59,86]. Pereira *et al.* discovered that 6 weeks after BDL, rats had higher serum creatinine levels and reductions in creatinine clearance, water excretion, and urinary sodium concentration; without the structural changes in the kidney that were features of HRS. The BDL rat at 4 weeks exhibited an intermediate stage of renal dysfunction. The authors suggested that BDL was a useful model to understand the pathophysiology of HRS [87]. HRS is the occurrence of renal failure in patients with advanced chronic liver disease, occasionally fulminant hepatitis, who have portal hypertension and ascites [88]. While Assimakopoulos *et al.* proposed that the BDL model was not appropriate for the study of the natural history of HRS because the renal impairment observed at the acute phase of the BDL model is based on a different pathophysiology than that of HRS, the chronic BDL model may be valid for the study of established HRS and its potential therapies [89].

In kidney, we found no significant differences in eNOS expression between rat with or without BDL. However, the BDL rat exhibited reduced renal expression of *n*NOS-α [59]. As in the liver, renal l-arginine and ADMA levels were higher in the BDL rat than sham control, but without alteration of ADMA/l-arginine ratios. In the kidney, SDMA concentrations were not different between shams and the BDL rat [50], which is due to the fact that SDMA is only removed via excretion while ADMA is mainly metabolized [90].

There is similar renal expression of DDAH-1 and DDAH-2 in the BDL and sham groups. Renal DDAH activity was significantly lower in the BDL group [50,59]. There was no significant difference in CAT-1 and -2 expression between BDL and sham groups [75,76].

Human studies also support the notion that plasma ADMA is increased in HRS. The levels of ADMA were higher in the cirrhotic patients with HRS than in those without this serious complication [91,92]. The levels of SDMA were also significantly higher in the patients with HRS compared to the patients without HRS [92]. Even in cirrhotic patients without HRS, the level of plasma ADMA was positively correlated with serum creatinine and negatively with creatinine clearance [80]. ADMA/l-arginine ratio was positively correlated with aspartate aminotransferase, creatinine and negatively with creatinine clearance [80].

*DDAH2* gene polymorphism is associated with chronic kidney disease and diabetes mellitus. Some variants of the *DDAH2* gene were reported to be associated with chronic kidney disease and insulin sensitivity. Sesti *et al.* reported that the rs9267551 functional variant of the *DDAH2* gene was related to chronic kidney disease. Carriers of the C allele have higher transcriptional activity resulting in increased expression of DDAH2 and lower plasma ADMA levels having a lower risk of renal dysfunction [93]. Moreover, Andreozzi *et al.* found the disposal of glucose was lower in GG carriers as compared with C carriers, which elaborated that a functional polymorphism of the *DDAH2* gene may confer increased risk for type 2 diabetes mellitus by affecting insulin sensitivity via increased ADMA levels [94].

### 9.3. ADMA and NO Dysregulation in the BDL Rat Brain

BDL-induced brain damage has been commonly used as a model of HE [95–98]. HE is defined as a spectrum of neuropsychiatric abnormalities in patients with liver dysfunction, after the exclusion of other known brain disease [99,100]. It is characterized by personality changes, intellectual impairment, and a depressed level of consciousness [99,100]. Both developing and adult BDL rats have spatial memory deficits [64,98].

Hyperammonemia is considered one of the main factors responsible for the neurological alterations found in HE. Recently, the relationship between hyperammonemia and altered brain NO signaling [101] and ADMA pathway [102] have been described. The glutamate-NO-cyclic guanosine monophosphate (cGMP) pathway is impaired in the brain of *in vivo* animal models of chronic moderate hyperammonemia and HE [103]. The impairment occurs at the level of activation of soluble guanylate cyclase by NO. The glutamate-NO-cGMP pathway plays an important role in the modulation of intracellular events and of intercellular communication, including long-term potentiation, a process underlying learning and memory [104,105]. It is believed that the impairment of this pathway may be responsible for some of the neurological alterations found in hyperammonemia and HE.

ADMA is involved in the pathophysiology of cerebrovascular disease [106] and NO is critically involved in spatial memory function [107]. Interestingly, epidemiological studies support a potential link between ADMA and cerebrovascular disease, and cognitive impairment, since both microangiopathy-related cerebral damage [108] and chronic renal failure [109] are associated with elevated ADMA levels as well as cognitive impairment [110]. Balasubramaniyan *et al.* demonstrated that brain ADMA levels were significantly higher in the rat 4 weeks after BDL and the ADMA values were reduced following treatment with ornithine phenylacetate. They also showed a marked abnormality in NO regulation in the cirrhotic rat brain, which could be restored by reducing ammonia concentrations using ornithine phenylacetate [102]. The ADMA/l-arginine ratio was increased [102], the brain PRMT-1 was decreased [95], and the DDAH-1 was reduced [102]. Likewise, Bajaj *et al.* found that patients with liver cirrhosis had poor cognition and higher serum ADMA [82].

### 9.4. Other Major Organ Involvement in the Multiple Organ Failure (MOF) Model and BDL in Terms of ADMA and NO Dysregulation

Richir *et al.* infused ADMA and arginase to increase plasma ADMA levels and decrease l-arginine levels in rat. They showed that low l-arginine plasma levels in combination with high ADMA plasma levels deteriorates systemic hemodynamics and suggested that diminished NO production may be involved in the onset of organ failure [111]. Perticone *et al.* reported that, even within the limits of the normal range, plasma l-arginine was higher in essential hypertensive than in normotensive subjects. They proposed that relatively higher l-arginine in essential hypertensives was a counter-regulatory response aimed at compensating NO inhibition by ADMA, a possibility supported by the direct relationship between plasma l-arginine and ADMA [112]. Moreover, the increased levels of ADMA cooperates with insulin resistance to increase cardiovascular risks in hypertensive patients [20,113]. Visser *et al.* found that the ADMA/l-arginine ratio is related to circulatory failure, organ failure and disease severity, and predicts mortality in shock patients. They proposed a pathophysiological mechanism in shock: the imbalance of l-arginine and ADMA contributes to endothelial and cardiac dysfunction resulting in poor organ perfusion and organ failure, thereby increasing the risk of death [114]. Koch *et al.* also stated that serum ADMA concentrations are significantly elevated in critically ill patients, associated with MOF and related to short- and long-term mortality risk [115]. Interestingly, O’Dwyer *et al.* designed a prospective observational study and demonstrated that the degree of acidemia and lactemia was directly correlated with ADMA levels in severe sepsis patients, and that the variant allele with G at position “-449” in the *DDAH II* gene was associated with increased ADMA concentrations [116]. Collectively, increased ADMA is critically involved in cardiovascular dysfunction in critical illness.

As reported by Ljubuncic *et al.*, BDL in the rat can result in increased systemic oxidative stress [61]. Increased oxidative stress may inhibit DDAH activity and lead to ADMA accumulation [65,68,117]. Elevated ADMA concentration is well known to be associated with major cardiovascular risk factors, such as hypertension and hypercholesterolemia [40]. Interestingly, BDL in the rat can cause cardiomyopathy [118,119]. Therefore, it is reasonable to suggest a role for increased ADMA in cardiomyopathy in BDL rats.

Breakdown of the intestinal barrier may increase intestinal permeability and allow movement of intraluminal contents across the mucosa, which can lead to MOF in critical illness [120,121]. Zhang *et al.* found that the gut barrier dysfunction was evident in patients with MOF compared with normal controls, and this change was more pronounced in non-survivors. Continuous blood purification cannot only improve general conditions, but can also improve gut barrier dysfunction that is associated with down-regulation of inducible NOS [121].

BDL in rat induced small intestine atrophy that included decreased villus density and mucosal thickness, and increased oxidative stress, which was characterized by increased intestinal lipid peroxidation, reduced glutathione, and increased glutathione disulfide and total non-protein mixed disulfides [122,123]. Similarly, malignant biliary obstruction patients had higher levels of intestinal oxidative stress [124] and cirrhotic patients had increased intestinal lipid peroxidation [125].

NO is also involved in intestinal injury in BDL rat [126,127]. Given that increased oxidative stress and NO homeostasis are involved in intestinal barrier disruption and intestine damage in the BDL rat, the role of increased circulatory ADMA on intestine damage in BDL rat needs further study.

## 10. Conclusions

The BDL rat exhibits cholestasis, increased systemic oxidative stress, increased circulatory and hepatic ADMA levels, and multiple organ damage. Given the similarity of increased circulatory ADMA and multiple organ damage, BDL can represent a model of increased circulatory ADMA with resultant multiple organ damage. Understanding the role and regulation of ADMA in major organs in the BDL rat has clinical implications to treat cholestatic liver disease and ADMA-related disorders.

## Abbreviations

ADMA: asymmetric dimethylarginine
BH_4_: tetrahydrobiopterin
BDL: bile duct ligation
CAT: cationic amino acid transporter
cGMP: cyclic guanosine monophosphate
DDAH: dimethylarginine dimethylaminohydrolase
eNOS: endothelial NOS
HE: hepatic encephalopathy
HRS: hepatorenal syndrome
iNOS: inducible NOS
MELD: Model for End-Stage Liver Disease
MOF: multiple organ failure
NO: nitric oxide
NOS: nitric oxide synthase
PRMT: protein arginine methyltransferase
SDMA: symmetric dimethylarginine
TIPS: transjugular intrahepatic portosystemic shunt
